# A case report of ectopic breast cancer of the left upper extremity with literature review

**DOI:** 10.3389/fonc.2025.1674230

**Published:** 2025-10-02

**Authors:** Shu-ni Jia, Dong Wang, Ting-ting Xue, Zhe-xia Zhao

**Affiliations:** ^1^ Department of Ultrasonography, Shanxi Bethune Hospital, Shanxi Academy of Medical Sciences, Third Hospital of Shanxi Medical University, Tongji Shanxi Hospital, Taiyuan, China; ^2^ Department of Interventional Therapy, Shanxi Province Cancer Hospital, Shanxi Hospital Affiliated to Cancer Hospital, Chinese Academy of Medical Sciences, Cancer Hospital Affiliated to Shanxi Medical University, Taiyuan, China

**Keywords:** ectopic breast cancer, case report, diagnosis, literature review, imaging examination

## Abstract

Ectopic breast cancer (EBC) is a rare type of breast cancer that originates from ectopic breast tissue. This article describes a case with left breast cancer complicated by a second primary breast cancer of the left upper extremity, and summarizes the relevant literature to review the epidemiology, clinical presentation, pathologic features, imaging tests, treatment strategies, and prognosis of EBC with the aim of providing clinicians with a comprehensive reference to improve the understanding and management of this disease.

## Introduction

Breast cancer is one of the most common malignant tumors among women worldwide. Ectopic breast cancer (EBC), as a specific type of breast cancer, has a low incidence (1, 2). The presence of ectopic breast tissue provides a potential “breeding ground” for breast cancer, and its rarity and complexity make it challenging to diagnose and treat. The most common site of ectopic breast tissue is the axilla, and there are also reports of EBC occurring in rare sites such as the face, neck, inguinal area, and vulva (3, 4). In this article, we report a case of EBC occurring in the left upper arm, and review the relevant literature in order to improve clinicians’ understanding of the clinical features, imaging characteristics, and key diagnostic points of this rare type of tumor.

## Case report

**Figure 1:**
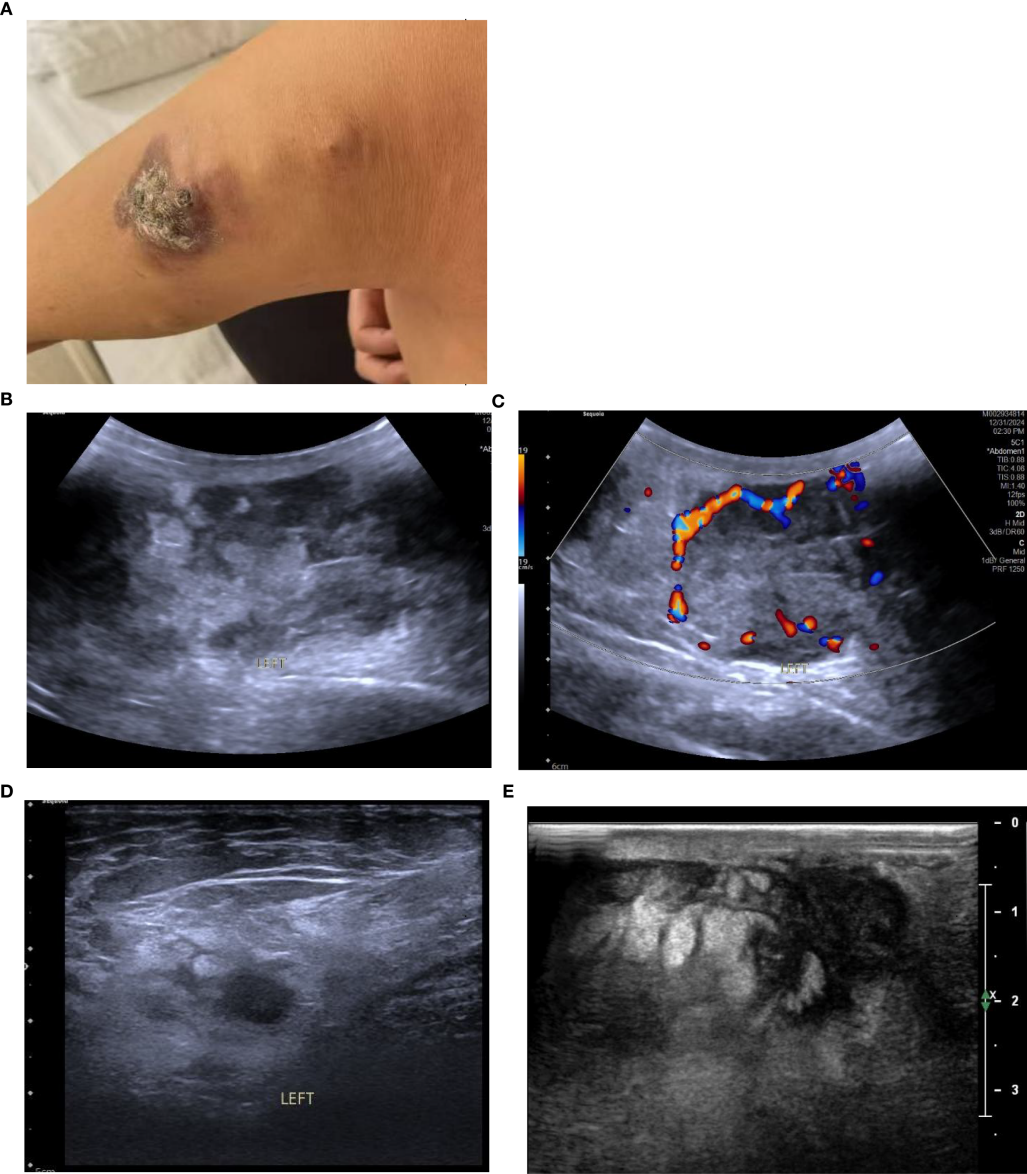
**(A)** The patient had a protruding red mass on the left upper limb, with a crust on the surface, hard in texture, ill-defined margins, and an irregular shape. **(B)** Conventional ultrasound revealed multiple hypoechoic solid masses in the subcutaneous tissue of the left upper arm, with ill-defined margins, indistinct edges, and irregular shapes; the largest measures 8.2 × 3.4 cm. **(C)** CDFI showed relatively rich blood flow signals within the masses. **(D)** Enlarged lymph nodes were visible in the left axilla, with clear margins and no obvious lymph node structure. **(E)** After two cycles of chemotherapy, the largest mass measures approximately 4.0 × 2.6 cm (a marked reduction from its pre-treatment size).

**Figure 2:**
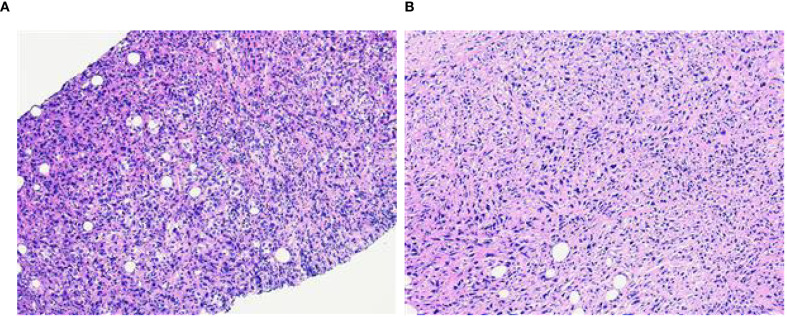
**(A)** The puncture pathology of the mass in the left upper limb showed that, in combination with the results of immunohistochemical staining, it was consistent with ectopic breast tissue, with some areas being tubular adenoma and some regions undergoing malignant transformation (metaplastic carcinoma) (HE×100). **(B)** The puncture pathology of the left axillary lymph node revealed a malignant tumor, which was considered to be a metastasis of the malignant component from the upper limb tumor (metaplastic carcinoma) (HE×100).

A 58-year-old female patient with no family history of breast cancer was found to have a left upper limb mass of about 2cm in size 30 years ago, which was not accompanied by redness, swelling and pain, so she did not pay attention to it and did not receive any treatment. During this period, the left upper extremity mass gradually increased to about 3 cm. In June 2024, the patient discovered a rapidly increasing mass in the left upper limb with a length of about 15cm, accompanied by pain, local redness, swelling, and ulceration. The patient sought medical attention at our hospital. Physical examination showed a raised red mass of about 15 × 8cm in the left upper limb, with scabs on the surface, hard texture, unclear boundaries, irregular shape, and obvious tenderness. (**Figure 1A**). Ultrasound examination showed multiple solid hypoechoic masses under the skin of the left upper arm, with unclear boundaries, blurred edges, irregular shapes, and abundant blood flow signals visible inside (**Figures 1B, C**). No obvious space occupying lesions were observed in both breasts. Multiple lymph node enlargement can be seen in the left axilla, with larger ones measuring 1.0 × 0.7cm in size, clear boundaries, regular morphology, and no obvious lymph node structure (**Figure 1D**). Ultrasound indicates multiple solid masses in the left upper arm, suggesting malignancy. Multiple lymph node swelling in the left axilla, suspected of metastasis. Chest CT shows multiple solid nodules in both lungs, with a high possibility of metastasis. The patient’s whole-body bone imaging, abdominal ultrasound, clavicle area ultrasound, and head MRI did not show any other tumor lesions. On January 2, 2025, the patient underwent ultrasound-guided biopsy of the left upper limb mass and left axillary mass. The pathological results of the left upper limb mass biopsy, combined with immunohistochemical markers, were consistent with ectopic breast tissue, with some areas being adenoma and others showing malignant transformation (metaplastic carcinoma). Immunohistochemistry results: ER (focal +), PR (focal weak +), HER-2 (0), Ki67 (approximately 80% +). PD-L1 CPS 80. The pathological results of the left axillary lymph node biopsy revealed malignant tumor, which was considered to be metastasis from the left upper limb malignant tumor (metaplastic carcinoma) (**Figure 2**).

Medical History:5 years ago, she underwent lumpectomy (breast-conserving surgery) for left breast cancer, and the postoperative pathology returned grade III invasive ductal carcinoma of the left breast (9 points), with immunohistochemistry: ER (+, about 80%), PR (+, about 90%), CerbB-2 (1+), and Ki67 (about 60%+). Intensive EC-P regimen chemotherapy was performed after surgery, and 4 cycles of EC regimen chemotherapy (epirubicin 130 mg + cyclophosphamide 850 mg) were completed from September 1, 2021 to October 12, 2021, and 4 cycles of P regimen chemotherapy (paclitaxel liposome 230 mg for injection) were performed from October 26, 2021 to December 8, 2021. At the end of chemotherapy, left breast and clavicular area, axillary and internal breast lymphatic drainage areas + tumor bed additive intensity-modulated radiotherapy was performed (PTV 50.40Gy/28 times, PGTVtb 65.80Gy/28 times). Since the completion of radiotherapy, the patient has been consistently taking letrozole for endocrine therapy.

Due to the patient’s refusal to undergo surgery, a combination of chemotherapy, radiotherapy, and endocrine therapy was used. Chemotherapy began on January 16, 2025. The specific medication was 200mg of albumin bound paclitaxel (Day 1 and Day 8) and 1.5g of capecitabine twice a day (Day 1 to Day 14), with a cycle of 21 days. The patient has completed two chemotherapy cycles and the left upper limb mass has shrunk compared to before (**Figure 1E**).

## Discussion

A total of 46 articles related to EBC from January 2005 to April 2025 in the PubMed database were retrieved and summarized together with this case. The general clinical and pathological data of the patient are shown in **Table 1**. The imaging manifestations of the patient are shown in **Table 2**. The collected data includes the patient’s gender, age, personal history, family history, location of occurrence, clinical manifestations, pathological type, metastasis, prognosis, and imaging findings. A total of 65 cases from 64 patients were collected (one case was bilateral axillary EBC). Because the integrity of clinical data obtained in each case was different, not every patient was included in the analysis of each variable.

**Table 1:** Clinical and pathological data of ectopic breast cancer cases published in the literature.

Characteristics	Total cases	Category	N	Incidence, %
Gender	66	Male	13	19.7
		Female	53	80.3
Presence of other malignancies	66	Yes	14	21.2
		Breast cancer	9	
		Thyroid cancer	1	
		Malignant melanoma of the thigh	1	
		Salivary gland adenocarcinoma	1	
		Hodgkin’s lymphoma	1	
		Colon cancer	1	
		No	52	78.8
Family history	27	Yes	7	25.9
		No	20	74.1
Site of occurrence	66	Axilla	48	72.7
		Vulva	9	13.6
		Chest wall	5	7.6
		Abdominal wall	2	3.0
		Inguinal region	1	1.5
		Upper limb	1	1.5
Clinical manifestations	61	Painless mass	22	36.1
		Tenderness	22	36.1
		Skin ulceration	8	13.1
		Skin erythema	8	13.1
		Asymptomatic	7	11.5
Tumor size	52	≤2cm	24	46.2
		>2cm, ≤5cm	20	38.5
		>5cm	8	15.4
Pathological type (definite)	52	DCIS	3	5.9
		IDC	37	72.5
		ILC	8	15.7
		Mucinous carcinoma	2	3.9
		Medullary carcinoma	1	2.0
Pathological type (undefined)	14	Adenocarcinoma only	11	
		Ductal carcinoma only	1	
		Adenocarcinoma with Paget’s disease	3	
Presence of distant metastasis	66	Yes	23	
		No	43	
ER	52	Positive	43	
		Negative	9	
PR	49	Positive	35	
		Negative	14	
CerbB-2	47	Positive	18	
		Negative	29	
Ki67	28	≤10%	7	
		>10%, ≤30%	11	
		>30%	10	

**Table 2:** Imaging data of reported cases of ectopic breast cancer in the literature.

Imaging modality	Total cases	Imaging features		Incidence, %
Ultrasound	20	Hypoechoic	20	100.0
		Heterogeneous echo	10	47.4
		Microcalcification	5	26.3
		Ill-defined margin	16	78.9
		Irregular margin (lobulation, angularity, spiculation)	18	89.5
		Irregular shape	18	89.5
		Aspect ratio > 1	8	42.1
		Rich blood supply	10	47.4
Mammography	7	Irregular shape	7	100.0
		Irregular margin	7	100.0
		High density	6	85.7
		Iso-density	1	14.3
	Calcification type	Amorphous calcification	1	14.3
		Coarse heterogeneous calcification	1	14.3
		Pleomorphic calcification	3	42.9
	Calcification distribution	Clustered	5	100.0
MRI	7	Signal: low T1, high T2	2	
		Irregular shape	3	
		Irregular margin	3	
		Enhancing mass	4	

### Epidemiology

The formation of ectopic breast tissue is related to the incomplete regression of the milk line during embryonic development. In the 6th week of the embryo, the milk line extends from the axilla to the inguinal region. Normally, the milk line will regress, leaving only the breast tissue in the chest. If the regression is incomplete, ectopic breast tissue may form (5). The incidence of ectopic breast tissue in the general population is approximately 0.22% to 6.0%, and it is more common in females, with a male-to-female ratio of about 1:5 (6). In this study, the average age of the patients was 55.7 ± 13.5 years (ranging from 27 to 83 years), including 13 males (20.0%) and 53 females (80.0%). The number of female patients was significantly higher than that of male patients, which is consistent with previous reports. Meanwhile, the age distribution of patients with EBC is similar to that of patients with breast cancer. In the cases collected in this study, 14 patients had other malignancies, among which breast cancer was the most common (accounting for 64.3%), indicating that the occurrence of EBC may be similar to the genetic and environmental risk factors of *in situ* breast cancer.

Literature reported that the risk of breast cancer increased with the number of first-degree relatives (mother, daughter, or sister) who had been diagnosed. Approximately 13% to 19% of breast cancer patients had affected first-degree relatives (mother, daughter, or sister). If one first-degree relative had breast cancer, the risk increased by 1.5 to 4 times (7). In this study, the proportion of patients with a first-degree relative having breast cancer was 25.9%, which was similar to the reported figures for breast cancer, indicating that family history was of great importance for patients. The most common site for ectopic breast cancer was the axilla, accounting for 70% to 90% of all EBC (8). Other possible sites included the chest wall, abdominal wall, vulva, neck, face, etc (3, 4, 8). In this study, 72.7% of EBC were located in the axilla, 13.6% in the vulva, 7.6% in the chest wall, 3.0% in the abdominal wall, and only 1 case was found in the inguinal region (1.5%). This case report presented a case of ectopic breast cancer occurring in the subcutaneous tissue of the left upper limb, for which no similar reports had been found in the literature so far.

### Clinical manifestation

The clinical manifestations of EBC were diverse. Common symptoms included palpable masses, skin erythema, ulcers, and pain (3, 9). Because ectopic breast tissue usually did not contain the nipple-areola complex and was located in special positions, these symptoms might have been mistaken for other diseases, such as lipomas, sebaceous cysts, or lymph node lesions (10). Moreover, EBC might not have had obvious symptoms in the early stage, leading to delayed diagnosis (11).

### Pathological features

Histopathological examination is the gold standard for the diagnosis of EBC. It is necessary to have the presence of both normal breast tissue and cancerous tissue to confirm a diagnosis of EBC (5, 9, 12). Immunohistochemical testing could serve as an auxiliary diagnostic tool, with commonly used markers including ER, PR, CerbB-2/HER2, and Ki67 (12–14).

The pathological features of EBC were similar to those of normal breast cancer and could manifest as a variety of histological subtypes. Infiltrating ductal carcinoma was the most common subtype (accounting for 72.5% in this article), followed by lobular carcinoma, medullary carcinoma, mucinous carcinoma, and so on (6, 9, 14). The histological diagnosis needed to take into account the presence of ectopic breast tissue and the morphological characteristics of the tumor, while also ruling out metastasis from breast cancer in other locations (13, 14). This case was diagnosed as metaplastic breast carcinoma (MBC). MBC is a rare and highly heterogeneous subtype of invasive breast cancer, accounting for 0.2–5% of all breast cancers. The tumor undergoes “metaplasia” from epithelial toward non-glandular lineages and may exhibit diverse components such as squamous, spindle, chondroid, or osseous elements. MBC typically presents as a large mass, behaves aggressively, and carries a high risk of local recurrence and pulmonary metastasis (15, 16). As with the normal breast, ectopic breast tissue could also undergo both benign and malignant pathological changes, that is, the progression from fibroadenoma or intraductal papilloma to carcinoma (5, 17). The pathological results reported in this case showed that the patient’s left upper limb mass was partially a tubular adenoma, with some areas having undergone malignant transformation (metaplastic carcinoma or myoepithelial carcinoma), and the pathological type of the left axillary lymph node tissue was consistent with that of the left upper limb mass, suggesting malignant transformation of ectopic breast adenoma with left axillary lymph node metastasis.

### Imaging examination

Ultrasound was the preferred diagnostic method for ectopic breast tumors. When the tumor was located in the breast line area and glandular-like echoes could be detected around it, it was helpful for the diagnosis of ectopic breast tumors. The criteria for determining benign or malignant tumors on ultrasound images were the same as those for breast tumors. Generally speaking, irregular morphology, unclear boundaries, rough or angular edges, and abundant blood flow signals were the ultrasound signs of malignant tumors. However, some ectopic breast tissues were located superficially, with thin glandular bodies and not in the breast line area, making it difficult to diagnose (4, 12, 18). In this case, the tumor was located in the left upper limb and there was no normal glandular echo around it, making it difficult to distinguish from other malignant tumors.

Sometimes, heterotopic breast cancer in the axilla could be displayed in the internal and external oblique mammography, but the rate of missed diagnosis was high, and heterotopic breast cancer in other locations could not be displayed (12, 18). In this article, a total of 7 patients were retrieved for mammography, and the location of EBC was the axilla, which was consistent with the X-ray findings of breast cancer. The findings included irregular shape, uneven edges, cluster distribution of polymorphic calcification, rough heterogeneous calcification, and amorphous calcification. These findings could be used as an auxiliary diagnostic method for ultrasound detection.

Magnetic Resonance Imaging (MRI) could reveal the characteristics of malignant tumors, such as irregular shape, uneven margins, and enhancement on contrast-enhanced scans (4, 6, 11). In addition, some patients underwent PET-CT scans, which could be used to assess the primary tumor, lymph node metastasis, and distant metastasis (9, 11, 13, 19).

### Treatment strategies

The treatment of most EBC was based on the standard treatment protocols for breast cancer (9, 10, 20). Surgery was the main treatment, supplemented by chemotherapy, radiotherapy, endocrine therapy, and molecular targeted therapy. Surgical treatment included local excision and lymph node dissection in the drainage area. Postoperative radiotherapy could reduce the risk of local recurrence, especially for patients with lymph node involvement or larger tumors. The indications for chemotherapy followed those for primary breast cancer and were usually determined based on the tumor stage and molecular subtype. For patients with hormone receptor-positive tumors, endocrine therapy could lower the risk of recurrence (9–12, 20–22). In addition, targeted therapy could be administered, with indications being the same as those for primary breast cancer. For example, patients with HER2-positive tumors could receive trastuzumab (20–22).

### Prognosis

The prognosis of EBC was worse than that of primary breast cancer due to its rarity and delayed diagnosis. However, recent studies had shown that the prognosis of EBC was not necessarily worse than that of primary breast cancer if it was diagnosed and treated at similar disease stages. The prognostic factors included tumor staging, lymph node involvement, and treatment plan.

## Conclusion

EBC is a rare but challenging disease that requires multidisciplinary collaboration, including radiology, pathology, surgery, and medical oncology. Increasing awareness of EBC, early diagnosis, and standardized treatment are key to improving patient prognosis. More research is needed in the future to further explore its pathogenesis and optimize treatment strategies.

## Data Availability

The original contributions presented in the study are included in the article/Supplementary Material. Further inquiries can be directed to the corresponding author.
